# COPD Patient’s Outcomes following Total Knee Arthroplasty—An Analysis of the National Inpatients Sampling (2016–2020)

**DOI:** 10.3390/jcm13185578

**Published:** 2024-09-20

**Authors:** Aviram Albagly, Ofer Kobo, Yaniv Yonai, Yaron Berkovich, Yaniv Steinfeld

**Affiliations:** 1 Orthopedic Surgery Department, Bnai Zion Medical Center, Haifa 31048, Israel; 2 Cardiology Division, Hillel Yaffe Medical Center, Hadera 38100, Israel; oferk@hymc.gov.il; 3 Orthopedic Surgery Department, Carmel Medical Center, Haifa 34361, Israel; yanivyonai@gmail.com (Y.Y.); yaronber@clalit.org.il (Y.B.); 4 Orthopedic Surgery Department, Mount Sinai Hospital, Toronto, ON M5G 1X5, Canada; yanivsteinfeld@gmail.com

**Keywords:** total knee arthroplasty, COPD, NIS, arthroplasty, surgical risk

## Abstract

Introduction: Total knee arthroplasty (TKA) is a common elective procedure aimed at improving patients’ quality of life. Patients undergoing this procedure can have a wide variety of comorbidities, including chronic obstructive pulmonary disease (COPD). Several studies demonstrated a higher risk of postoperative complications for this patient population. In this study, we examined the mortality risk of this group of patients, as well as the length of stay (LOS) and general costs. Methods: This study is a retrospective, case–control study. Using the National Inpatients Sampling (NIS) database, we defined a cohort of adults who received their inpatient primary TKA between 1 January 2016 and 31 December 2020. Preoperative variables include age, sex, race, primary payer, hospital location, and hospital type. Outcomes examined in this study include overall patient mortality as a primary outcome. Secondary outcomes include total LOS (in days) and inpatient costs in the United States (in USD). Results: A total of 2,835,499 patients who underwent TKA procedure in the United States were included. A total of 173,230 (6.1%) COPD patients were included in the COPD group. The mortality rate in the COPD group (0.1%) was more than three times higher than the control group (0.03%, *p*-value < 0.001). Patients in the COPD group had a longer in-hospital length of stay (2.76) compared to the control group (2.31, *p*-value < 0.001) and a higher treatment cost (average value of treatment per patient) (USD 69,386) compared to the control group (USD 64,446, *p*-value < 0.001). We also found higher mortality risk for patients older than 60 and patients of white ethnicity. Conclusion: COPD patients undergoing TKA have a higher mortality rate and this issue should be addressed in order to improve patient care and outcomes.

## 1. Introduction

Total knee arthroplasty (TKA) is a common procedure designated to improve a patient’s quality of life and mobility. In 2010, the prevalence of TKA in the total U.S. population was 1.52% [1]. In 2018, a retrospective review was held using the NIS, based on 2000–2014 data, anticipating that primary TKA is projected to grow by 85%, to 1.26 million procedures, by 2030 [2].

Degenerative joint disease (DJD) has many comorbidities and predisposing risk factors. Particularly, although the reason remains somewhat unclear, there is a positive association between chronic obstructive pulmonary disease (COPD) and DJD [3,4]. Some studies describe age-related changes in COPD patients’ bones, bone mass decline, and even osteoporotic changes [5,6].

COPD is a common disease, affecting 2.4% of the global population as of 2015 [7]. The prevalence of COPD in the United States is estimated to be between 14 and 17% [8], and remains one of the leading causes of death in the USA, with age-adjusted mortality rates of 39.7 per 100,000 persons between the years 2014 and 2015 [9].

Mortality rates after TKA procedures are relatively low, and risk increases as the patients have more comorbidities. A systematic review carried out in 2018 demonstrated a 0.39% 90-day mortality rate [10].

There is a positive association between COPD and mortality after general surgeries. Sankar et al. [11] found that COPD patients have decreased survival and increased costs in the year after general surgeries. Analyzing almost one million patients who had undergone general surgeries, including orthopedic ones, 18% of them had COPD; this condition was associated with a 61% increase in hazard of death and 13% greater total costs after adjustment for sociodemographic factors and procedure type.

Moreover, a previous study estimated an astonishing 35% increase in odds of morbidity and a 30% increase in odds of death which, after risk adjustment, were attributed to COPD [12].

With regard to the specific outcomes of COPD patients after TKA procedures, Klasan et al. [13] examined the overall risk of major complications for COPD patients following TKA and found a significantly higher risk of postoperative pneumonia and wound infections, as well as longer in-hospital length of stay and readmission rates. Vakharia et al. [14] also found a higher risk of venous thromboembolism following TKA in those patients. Other studies also found that patients with COPD are more likely to experience short- and long-term complications following total joint arthroplasties, including pneumonia, re-intubation, cardiac arrest, and deep infection [15,16]. Pulmonary complications still count as one of the leading causes of postoperative complications in COPD patients. Data show that COPD is associated with an increased risk of postoperative pneumonia and respiratory failure [12,13].

As the number of TKA procedures is rapidly growing in the U.S. population, as is COPD prevalence, it is crucial to examine how COPD will alter the outcomes in order to optimize our understanding, to better explain this risk factors and its consequences for TKA patients, and to highlight the importance of careful risk prediction and decision-making for patients with COPD who are considering this procedure.

In this study, we aim to examine and compare the outcomes of COPD and non-COPD patients undergoing TKA procedures, according to the National Inpatient Sampling (NIS) database. The primary objective was in-hospital mortality and the secondary objectives included the length of stay (LOS) and costs.

Our hypothesis is that COPD will, in fact, alter the outcomes in TKA procedures, resulting in a higher mortality rate, higher length of stay (LOS), and increased cost per patient.

## 2. Methods

### 2.1. Study Sample

This is a retrospective case–control study. We defined a cohort of adults who received their primary TKA between 1 January 2016 and 31 December 2020.

### 2.2. Data Source

We extracted data from the NIS database from 2016 to 2020 on all inpatient hospitalizations with associated coding for TKA procedures. Inclusion criteria were patients who could be identified using the International Classification of Diseases (ICD-10) procedure codes (OSRC, OSRD). We then categorized the cohort into the presence of COPD using ICD-10 coding (J441, J449).

### 2.3. Outcomes

Preoperative variables included age, sex, race, primary payer, hospital location, and hospital type. The data elements that were examined in this study included overall inpatient mortality as a primary outcome during the initial hospitalization. Secondary outcomes included the total length of stay (LOS, in days) and inpatient costs in the United States (in USD).

The NIS is a de-identified database and is publicly available, so this study was exempt from IRB approval.

### 2.4. Statistical Analysis

All statistical analyses were conducted using the SPSS statistical analysis software (IBM, version 29.0). Patients’ characteristics were categorized by the presence or absence of COPD. Chi-squared tests were used for categorical variables and *t*-tests were used for continuous variables. A *p*-value < 0.05 was considered statistically significant for all the tests. Adjusted odds ratios and their corresponding 95% confidence intervals were used for measuring predictors of mortality in patients with COPD.

## 3. Results

A total of 2,835,499 patients who underwent TKA procedures in the United States between the years 2016 and 2020 were collected, identified, and included in the primary analysis. Within that cohort, 2,662,269 patients did not have a COPD diagnosis in their medical history and were thus referred to as the control group. A total of 173,230 (6.1%) COPD patients were included in the COPD group.

Table 1 demonstrates the demographic characteristics of these patients in detail. COPD patients were more likely to be female (60.9%, *p*-value < 0.001) compared to male (39.1%) and were older than the control group (68.01 y.o and 66.85 y.o, respectively, *p*-value < 0.001). The vast majority of the population who underwent TKA in both the COPD group and the control group were of white ethnicity (82.2% and 77.7%, respectively, *p*-value < 0.001). A total of 70.1% of the COPD group and 56.3% of the control group used Medicare as their primary payer, more than any other medical financial option in the United States.

The characteristics of the included hospitals are also shown in Table 1. Approximately two-thirds of both the COPD group and the control group (59.3% and 63%, respectively, *p*-value < 0.001) went to urban teaching hospitals, and the majority of the included hospitals were private, not-for-profit facilities (72.4% of the COPD group, 74.1% of the control group, *p*-value < 0.001).

Results of the analysis of the outcomes of both the COPD and control groups after TKA are shown in Table 2. Inpatient mortality rates in the COPD group were more than three times higher than the control group, with 0.1% (170 out of 173,230) inpatient mortality in the COPD group compared to 0.03% (830 out of 2,662,269) in the control group (*p*-value < 0.001).

Patients in the COPD group had a longer in-hospital length of stay compared to the control group, with a mean of 2.76 days (2.21 SD) compared to a mean of 2.31 days (SD 1.91) for the control group (*p*-value < 0.001).

The average total patient costs (in USD) for the COPD group were higher, with a mean of USD 69,386 (SD USD 50,336) than that of the control group, with a mean of USD 64,446 (SD USD 45,501). All the data were statistically significant, with a *p*-value < 0.001.

A subsequent multivariant analysis for predictors of mortality in patients with COPD reveals that positive predictors for mortality included older age, above 60 years old, with an adjusted odds ratio (OR) of 4.034 (95% CI 2.125–7.658, *p*-value < 0.001), and white ethnicity, with an adjusted OR of 5.123 (95% CI 2.101–12.490, *p*-value < 0.001). In addition, there was a statistically insignificant trend showing that mortality was higher in nongovernmental hospitals compared to governmental. An analysis of gender revealed no statistical differences between males and females. Table 3 summarizes the results for predictors of mortality in COPD patients.

## 4. Discussion

In this study, we analyzed the outcomes of TKA in COPD patients using the NIS database between the years 2016 and 2020. We found that compared to the control group, COPD patients had a significantly higher inpatient mortality (approximately three times higher), longer inpatient length of stay, and higher costs per patient. In addition, we found increased mortality risk associated with older age above 60 years old and white ethnicity. Analysis of gender and hospital type revealed no statistical significance.

This increase in inpatient mortality can be explained by the higher risk of postoperative complications.

One of the main concerns regarding postoperative complication for COPD patients are pulmonary complications. In the lungs of patients with COPD, the chronic hypoxia caused by airflow limitation increases the activity of angiotensin-converting enzyme (ACE), which can further impair peripheral oxygen utilization and respiratory muscle function. Additionally, activation of the renin–angiotensin system (RAS) may result in cell proliferation, hypertrophy, vasoconstriction, and inflammation of the pulmonary vasculature [17].

Furthermore, previous studies demonstrated a higher risk of systemic and nonsystemic postoperative complications in COPD patients [13,15,16], such as cardiac complications, renal complications, and superficial site infections and sepsis.

Cardiac complications may occur due to surgical stress increasing the myocardial oxygen demand, which in the already limited airflow of COPD patients can lead to postoperative myocardial infraction [17].

Another postoperative risk factor for COPD patients is an increased risk of stroke. It is believed that the use of anticholinergics and beta-agonists by patients with COPD may possibly increase the risk of stroke by suppressing parasympathetic control for the former and stimulating sympathetic control for the latter [18].

In addition, COPD patients tend to have higher rates of venous thromboembolism following primary TKA [14]. This can be explained by systemic inflammation as the main atherothrombotic abnormality in COPD, along with other factors that may play a role, such as hypoxia-related platelet activation or oxidative stress [19].

In this study, we did not address technical operative issues in COPD patients. We do believe that low bone quality in COPD patients (related to various factors but mainly long-term corticosteroid use) can increase postoperative complications, procedure-related morbidity, and, eventually, mortality.

This study has several limitations that need to be addressed. The constitutional limitations within the NIS database include, among others [20], a lack of clinical granularity and a lack of outcome data other than inpatient events. In addition, we were unable to determine the severity index score of COPD (GOLD criteria); thus, we were unable to differentiate the inpatient mortality according to the severity of the COPD in the group. Moreover, morbidity and mortality events that were possibly related to the surgical procedure and occurred on an outpatient basis were not documented in this database.

Another limitation is not including the outpatient TKA patients in the database. While we do not have an estimation of the number of cases, we assume that the patients selected for “same-day discharge” are healthier than the general population. We assume that the mortality rate in those patients is lower, thus making the mortality risk higher in the COPD group.

Another limitation is that we could not differentiate the type of anesthesia used in the surgery. It is well known that general anesthesia adds morbidity and mortality risk for COPD patients, but, unfortunately, we do not have the data regarding its use.

## 5. Conclusions

Patients with COPD who underwent TKA have poorer outcomes, with a three times higher mortality rate, longer LOS, and increased cost compared to non-COPD patients who underwent the same procedure. Furthermore, older age above 60 years old and white ethnicity constitute additional risk factors for inpatient mortality among COPD patients. Given the high prevalence of COPD patients in the United States and the world generally, and the rapid anticipated growth of TKA procedures, it is crucial to address these patients’ specific risk factors in order to optimize their outcomes and lower the risk of postoperative complications and mortality. We recommended making a team assessment for each patient individually for their operative and postoperative risk and matching the correct postoperative treatment and follow-up, thus lowering the morbidity and mortality risk of this elective procedure.

## Figures and Tables

**Table 1:** Baseline cohort characteristics.

	*p*-Value	Total (N)	COPD	Non-COPD
Procedure type:				
TKA:	2,662,269 (93.9%)	173,230 (6.1%)	2,835,499	
Age at admission:				
N:	2,662,219	173,230	2,835,449	<0.001 ^1^
Missing:	50	0	50	
Mean:	66.58	68.01	66.67	
Median:	67	68	67	
Gender:				
Total:	2,661,730	173,190	2,834,920	<0.001 ^2^
Missing:	540	40	580	
Male:	1,027,150 (38.6%)	67,655 (39.1%)	1,094,805 (38.6%)	
Female:	1,634,580 (61.4%)	105,535 (60.9%)	1,740,115 (61.4%)	
Race:				
Total:	2,556,024	167,715	2,723,739	<0.001 ^2^
Missing:	106,245	5515	111,760	
White:	2,068,734 (77.7%)	142,385 (82.2%)	2,211,119 (78%)	
Black:	212,665 (8%)	14,135 (8.2%)	226,800 (8%)	
Hispanic:	164,195 (6.2%)	6480 (3.7%)	170,675 (6%)	
Asian/Pacific Islanders:	40,770 (1.5%)	1115 (0.6%)	41,885 (1.5%)	
Native American:	11,980 (0.4%)	870 (0.5%)	12,850 (0.5%)	
Other:	57,680 (2.2%)	2730 (1.6%)	60,410 (2.1%)	
Primary payer:				
Total:	2,658,990	173,075	2,832,065	<0.001 ^2^
Missing:	3280	155	3435	
Medicare:	1,499,785 (56.3%)	121,385 (70.1%)	1,621,170 (57.2%)	
Medicaid:	110,660 (4.2%)	13,115 (7.6%)	123,775 (4.4%)	
Private:	948,035 (35.6%)	32,295 (18.6%)	980,330 (34.6%)	
Uninsured:	13,320 (0.5%)	620 (0.4%)	13,940 (0.5%)	
No charge:	1165 (0.0%)	50 (0.0%)	1215 (0.0%)	
Other:	86,025 (3.2%)	5610 (3.2%)	91,635 (3.2%)	
Hospital location:				
Total:	2,662,269	173,230	2,835,499	<0.001 ^2^
Rural:	262,924 (9.9%)	23,450 (13.5%)	286,374 (10.1%)	
Urban, non-teaching:	721,960 (27.1%)	47,055 (27.2%)	769,016 (27.1%)	
Urban, teaching:	1,677,384 (63%)	102,725 (59.3%)	1,780,109 (62.8%)	
Hospital type:				
Total:	2,662,269	173,230	2,835,499	<0.001 ^2^
Governmental:	222,424 (8.4%)	15,365 (8.9%)	237,789 (8.4%)	
Private, non-profit:	1,973,850 (74.1%)	128,600 (72.4%)	2,102,450 (74.1%)	
Private, invest-owned:	465,996 (17.5%)	29,265 (16.9%)	495,261 (17.5%)	

^1^ Independent *t*-test. ^2^ Chi-squared test.

**Table 2:** Outcomes after TKA.

	COPD	Non-COPD	*p*-Value
In-hospital mortality:	170 (0.1%)	830 (0.03%)	<0.001 ^2^
LOS (days):			
Mean (SD):	2.76 (2.21)	2.31 (1.91)	<0.001 ^1^
Median (IQR):	2.00 (2.00–3.00)	2.00 (1.00–3.00)	
Cost (USD):			
Mean (SD):	69,386.73 (50,336.00)	64,446.35 (45,501.87)	<0.001 ^1^
Median (IQR):	56,576.61 (40,104.00–83,104.00)	53,480 (38,426.00–77,107.00)	

^1^ Independent *t*-test. ^2^ Chi-squared t.

**Table 3:** Multivariant analysis for predictors of mortality in patients with COPD.

Variable	Comparison	Adjusted OR	95% CI		*p*-Value
Age:	Age above 60 y.o	4.034	2.125	7.658	<0.001
Sex:	Female sex	1.022	0.745	1.401	0.895
Race:	White ethnicity	5.123	2.101	12.490	<0.001
Hospital type:	Govermental	1.511	0.946	2.416	0.084

## Data Availability

The original data presented in the study are openly available from HCUP Databases. Healthcare Cost and Utilization Project (HCUP). June 2024. Agency for Healthcare Research and Quality, Rockville, MD. https://hcup-us.ahrq.gov/nisoverview.jsp (13 August 2024).
